# Association between *UGT1A1* Polymorphism and Risk of Laryngeal Squamous Cell Carcinoma

**DOI:** 10.3390/ijerph13010112

**Published:** 2016-01-07

**Authors:** Hui Huangfu, Hong Pan, Binquan Wang, Shuxin Wen, Rui Han, Li Li

**Affiliations:** 1 Department of ear-nose-throat (ENT), the First Hospital of Shanxi Medical University, 85 Jiefangnan Road, Taiyuan, 030001 Shanxi, China; 13906641499@163.com (H.P.); wbq_xy@126.com (B.W.); wensxsx@163.com (S.W.); bertie2@sina.com (R.H.); 2 Department of biology, the Basic Medical School of Shanxi Medical University, 56 Xinjiannan Road, Taiyuan, 030001 Shanxi, China

**Keywords:** laryngeal squamous cell carcinoma, genetic polymorphism, *UGT1A1*, high resolution melting curve

## Abstract

Laryngeal cancer is one of the largest subgroups of head and neck cancers. In addition to smoking and alcohol consumption, genetic polymorphisms are also risk factors for the development of laryngeal cancer. However, the exact relation between genetic variants and pathogenesis of laryngeal cancer has remained elusive. The aim of this study was to examine UGT1A1*6 (rs4148323 A/G) polymorphisms in 103 patients with laryngeal cancer and 220 controls using the high resolution melting curve (HRM) technique and to explore the association between UGT1A1*6 (rs4148323 A/G) polymorphisms and laryngeal cancer. The results showed an association between the rs4148323 G allele and increased risk of laryngeal cancer. While there was no statistically significant difference between rs4148323 genotype frequencies and different histological grades or different clinical stages of laryngeal cancer, stratification analysis indicated smoking or alcohol consumption and rs4148323 G allele combined to increase the risk of laryngeal cancer. In conclusion, the rs4148323 G allele is associated with the high *UGT1A1* enzyme activity, and might increase the risk of laryngeal cancer. Furthermore, smoking or alcohol consumption and the rs4148323 G allele act synergistically to increase the risk of laryngeal cancer.

## 1. Introduction

Laryngeal cancer is one of the largest subgroups of head and neck cancers. It is considered a serious public health problem in many countries, including China. Smoking and heavy chronic alcohol consumption are considered to be the most important risk factors for development of laryngeal cancer. However, differences in susceptibility among individuals influence the risk of developing a smoking- or alcohol consumption-related cancer; therefore, the exact pathogenesis of laryngeal cancer has remained elusive [1,2,3].

Uridine diphosphate glucuronosyltransferase enzymes (UGTs) catalyze the conjugation of many compounds with Uridine diphosphate -glucuronic acid, which are subsequently excreted via bile or urine. Uridine diphosphate glucuronosyl transferase 1 family polypeptide A1 (*UGT1A1*) is located on chromosome 2q37, and encodes a part of the UGT enzyme. The UGT1A1 enzyme is an important UGT involved in the detoxification of tobacco smoke carcinogens, like benzopyrenes [4,5,6] and genetic polymorphisms in UTG1A1 are associated with the risk of head and neck cancer [7,8]. Moreover, the preferred substrate for the UGT1A1 is bilirubin, which catalyzes the glucuronidation of bilirubin and facilitates the excretion of bilirubin [9]. Several studies have reported that bilirubin was a potent antioxidant, and hypothesized that it might play a protective role against cancer [10,11,12]. Serum concentrations of bilirubin were inversely correlated with UGT1A1 activity [13,14,15,16,17], indicating that low activity might increase bilirubin levels, with a corresponding decrease in the risk of developing cancer. Lacko *et al.* [7] showed that UGT1A1*28 polymorphisms were associated with a decreased enzyme activity, increased serum bilirubin levels and reduced risk of developing head and neck cancers. Genetic polymorphisms in UGT1A1*6 rs4148323 were reported to be associated with the UGT1A1 enzyme activity, and the rs4148323 G > A decreased the enzyme activity [18,19]. In this study, we investigated the UGT1A1*6 polymorphism in patients with laryngeal cancer by means of HRM technique, as well as smoking or alcohol consumption in the occurrence of laryngeal cancer.

## 2. Experimental Section

A total of 103 patients (99 men and four women, median age 65 years, range 43 to 82 years) and 220 age- and sex-matched healthy donors (211 men and nine women, with median age 63 years, range 40 to 78 years,) were enrolled in this study. All of them were permanent residents of the Shanxi area in China. All patients with laryngeal cancer were treated in the Ear-Nose-and-Throat (ENT) Department of the First Hospital of Shanxi Medical University and the Department of Head and Neck Surgery of Shanxi Cancer Hospital from January 2013 to February 2015. A diagnosis of laryngeal squamous cell carcinoma was confirmed by endoscopic pathology in all cases. According to the growth and severity of the tumor invasion, and the 2002 edition of the International Anti-Cancer Association (UICC) TNM standards, 103 patients were divided into five stages: stage 0 (2 cases); stage I (30 cases); stage II (38 cases); stage III (27 cases); stage IV (sixcases). We collected information on tobacco use and alcohol consumption for all cases and controls. Smoking was calculated as the sum of cigarettes according to [20] (20 cigarettes = 1 pack, a pack/day for 1 year = 1 pack-year). Ever-smokers were defined as subjects who smoked ≥ 1 pack-year, while never-smokers were defined as subjects who had never smoked or subjects who smoked < 1 pack-year but had quit smoking for more than 6 months. Alcohol consumption was calculated according to the literature [21], where one drink was defined 12.9 grams of ethanol (30 grams of liquor). Ever-drinkers were subjects who had intake ≥1 drink per day for more than 10 years. Never-drinkers were subjects who had never drunk or whose alcohol consumption<1 drink/day and had given up drinking for more than 6 months. Clinical data were collected from patient charts for all laryngeal cancer subjects recruitedinto the study.

Peripheral blood samples (2 mL) were collected from all subjects at the time of diagnosis. Blood genomic DNA was extracted using a whole blood genomic DNA extraction kit (TIANGEN, Beijing, China). The rs4148323A/G polymorphism was investigated by HRM. Primers used for PCR-HRM analysis of small amplicons were as follows: F: ACCTGACGCCTCGTTGTA; R: AATGGCACAGGGTACGTCTTC. PCRs were carried out in a total volume of 10 μL containing 1.0 μL of genomic DNA, 0.1 μL of forward and reverse primers (10 pmol/μL) each, 5 μL of 2 × PCR Premix Taq enzyme (TaKaRa: Kyoto, Japan), 0.2 μL high and low temperature calibrator mix, 1.0 μL LC Green Saturated fluorescent dye (Idaho Technology Inc.: Salt Lake City, UT, USA), and distilled water was added to give a final volume of 10 μL. PCR conditions were as follows: initial denaturation at 95 °C for 5 min, followed by 35 cycles of denaturation at 95 °C for 30 s, annealing at 53 °C for 30 s, and extension at 72 °C for 8 s, with a final incubation at 72 °C for 7 min. After two cycles of 95 °C for 30 s and 24 °C for 4 min, PCR products were genotyped automatically using a LightScanner™ Instrument 96 (Idaho Technology Inc.). Four samples were randomly selected from the three verified genotypes of each curve, amplified by PCR and sequenced at Sangon Biotech (Shanghai, China) to confirm rs4148323 genotyping results.

### 2.1. Statistical Analysis

Statistical analyses were performed using SPSS 17.0 (SPSS Inc.: Chicago, IL, USA). Pearson’s chi-square test was used to test the Hardy-Weinberg equilibrium for rs4148323 SNPs among controls using a cutoff of *p >* 0.05. Odds ratio (ORs) and 95% confidence intervals (CIs) were calculated to compare genotype frequencies between laryngeal cancer cases and controls using Pearson’s chi-square tests or Fisher’s exact test. *p* < 0.05 was considered to indicate statistical significance.

### 2.2. Ethical Considerations

The study protocol has been approved by the Ethics Committee of the Shanxi Medical University (2012006) and was conducted in accordance with the Declaration of Helsinki. Written informed consent was obtained from all participants and the legal guardians in case of minors of this study.

## 3. Results

The characteristics of the participants were summarized in Table 1. There were no significant difference of age and sex in laryngeal cancer cases and the controls (*p* > 0.05). There were more smokers and alcohol consumption in laryngeal cancer cases than in controls (*p* < 0.001).

**Table 1:** The characteristics of participants.

Variables	Laryngeal Cancer Cases (*n* = 103) N (%)	Controls (*n* = 220) N (%)	*p*-Value ^a^
**Demographic characteristics**			
**Gender**			
Male	99 (96.12)	211 (95.91)	
Female	4 (3.88)	9 (4.09)	1.000
**Age**			
<60	25 (24.27)	64 (29.09)	
≥60	78 (75.73)	156 (70.91)	0.366
**Clinical stages**			
0	2 (1.94)		
I	30 (29.13)		
II	38 (36.89)		
III	27 (26.21)		
IV	6 (5.83)		
**Histological grades**			
Low-differentiation	26 (25.24)		
Moderate-differentiation	40 (38.83)		
High-differentiation	37 (35.92)		
**Exposure Status**			
Smoking status			
Never-smokers	22 (21.36)	108 (49.09)	
Ever-smokers	81 (78.64)	112 (50.91)	<0.001
**Alcohol consumption**			
Never-drinkers	36 (34.95)	134 (60.91)	
Ever-drinkers	67 (65.05)	86 (39.09)	<0.001

^a^ Pearson’s chi-square test.

**Figure 1:**
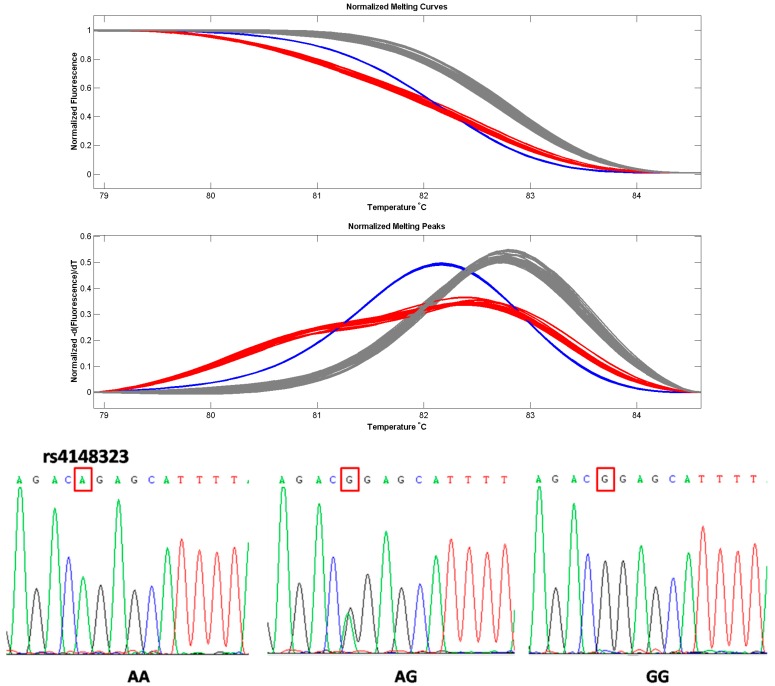
Genotyping and sequencing of rs4148323. Melting curves and genotyping results showed AA genotypes (blue curves), AG heterozygous types (red curves) and GG genotypes (gray curves). Sequencing results confirmed homozygotes (AA), heterozygotes (AG) and homozygotes (GG).

The rs4148323 SNP locus of UGT1A1*6 was successfully genotyped using HRM. Sequencing results were consistent with the genotypes of variants identified by high resolution melting curves (Figure 1). The distribution of rs4148323 SNP loci genotypes did not deviate significantly from the Hardy-Weinberg equilibrium in the control group (*p* > 0.05). The genotype frequencies distributions of *UGT1A1* rs4148323 polymorphisms in laryngeal cancer cases and controls were shown in Table 2.

**Table 2:** The genotype frequencies of rs4148323 in laryngeal cancer patients and controls.

Genotype	Patients (*n* = 103) Number (%)	Controls (*n* = 220) Number (%)	OR (95% CI) ^a^	*p*
rs4148323				
AA	6 (5.8)	29 (13.2)	1.00 (reference)	
AG	21 (20.4)	62 (28.2)	1.142 (0.893–1.460)	0.335
GG	76 (73.8)	129 (58.6)	1.135 (1.032–1.249)	0.022 *

^a^ Pearson’s chi-square test; * *p* < 0.05.

The genotype distribution of rs4148323 polymorphisms showed that the proportion of homozygous G/G was significantly higher in laryngeal cancer cases than in the controls (*p* < 0.05). The homozygous G/G was associated with increased risk for developing laryngeal cancer (OR = 1.135; 95% CI 1.032–1.249) in this study cohort. Allele frequencies analysis revealed that the rs4148323 G allele was associated with laryngeal cancer (OR = 1.149; 95% CI 1.052–1.254). The minor allele frequencies (MAF) of rs4148323 in different races are also shown in Table 3. In this SNP, MAFs of Asians were similar, and those of Han Chinese was higher than those in other ethnic groups, while those in European American and African American subjects were significantly lower than those in Asians.

**Table 3:** The allele frequencies in laryngeal cancer patients and controls and the MAF of rs4148323.

rs4148323 Allele	Patients (Gene Frequency)	Controls (Gene Frequency)	OR (95% CI) ^a^	*p*	MAF [22]			
					EA or AA	KR	JP	HC
A	0.16	0.27	1.00 (reference)		-	0.172	0.106	0.223
G	0.84	0.73	1.149 (1.052–1.254)	0.004 *				

^a^ Pearson’s chi-square test; MAF, minor allele frequency; EA, European American; AA, African American; KR, Korean; JP, Japanese; HC, Han Chinese; * *p* < 0.01.

The genotype frequencies of rs4148323 in different histological grades (highly differentiated, moderately and low differentiated) and different clinical stages of laryngeal cancer cases were analyzed. The results showed that there were no associations between rs4148323 polymorphism and different histological grades or different clinical stages of laryngeal cancer (*p* > 0.05, Table 4).

**Table 4:** The genotype frequencies in different histological grades and clinical stages of laryngeal cancer cases.

Patients	AA Number (%)	AG+GG Number (%)	OR (95% CI) ^a^	*p*
**Histological grade**				
Moderate or low-differentiation	3(4.6)	63 (95.5)	1.0 (reference)	
High-differentiation	3 (8.1)	34 (91.9)	1.039 (0.931–1.159)	0.664
**Clinical stage**				
0–II	4 (5.7)	66 (94.3)	1.00 (reference)	
III–IV	2 (6.1)	31 (93.9)	1.004 (0.904–1.114)	1.000

^a^ Fisher’s exact test.

Stratification analysis of the association of the rs4148323 polymorphism with tobacco smoking habits, alcohol consumption was performed. The rs4148323 A/G+G/G combined genotypes were associated with a higher risk of laryngeal cancer among tobacco smokers (OR = 1.109, 95% CI 1.013–1.214) and alcohol consumers (OR = 1.175, 95% CI 1.057–1.306) (Table 5).

**Table 5:** Stratification analysis of the strength of the association between *UGT1A1* rs4148323 and laryngeal cancer.

Variables	Patients/Control	AA	AG+GG	*p*	OR (95% CI) ^a^
**Smoking**					
Ever	81/112	4/16	77/96	0.035 *	1.109 (1.013–1.214) ^a^
Never	22/108	2/13	20/95	1.000	1.033 (0.890–1.200) ^b^
**Drinking**					
Ever	67/86	2/14	65/72	0.008 **	1.159 (1.046–1.284) ^a^
Never	36/134	4/15	32/119	1.000	1.001 (0.879–1.140) ^b^

^a^ Pearson’s chi-square test; ^b^ Fisher’s exact test; * *p* < 0.05; ** *p* < 0.01.

Finally, we analyzed the interaction between rs4148323 polymorphisms and smoking or drinking. The results showed that smoking and the rs4148323 G allele combined to increase the risk of laryngeal cancer (OR = 1.563, 95% CI 1.314–1.858). The same synergistic effect was found in relation to alcohol consumption and the rs4148323 G allele (OR = 1.784, 95% CI 1.416–2.247) (Table 6).

**Table 6:** The interaction between smoking or drinking and rs4148323 polymorphisms.

Variables	Genotype	Patients	Control	OR (95% CI)	*p*
**Smoking**					
Never	AA	2	13	1.00 (reference)	
Ever	AA	4	16	1.208 (0.628–2.324) ^a^	0.680
Never	AG+GG	20	95	1.00 (reference)	
Ever	AG+GG	77	96	1.579 (1.327–1.879) ^b^	<0.001
**Drinking**					
Never	AA	4	15	1.00 (reference)	
Ever	AA	2	14	0.690 (0.210–2.276) ^a^	0.666
Never	AG+GG	32	119	1.00 (reference)	
Ever	AG+GG	65	72	1.778 (1.413–2.237) ^b^	<0.001

^a^ Fishr’s exact test; ^b^ Pearson’s chi-square test.

## 4. Discussion

In this study on the relation between the UGT1A1*6 polymorphism and the risk of laryngeal cancer, we found an association between the rs4148323 G allele and an increased risk of laryngeal cancer. The genotype frequencies of rs4148323 had no association with different histological grades or different clinical grades of laryngeal. The results also indicated that smoking or alcohol consumption and the rs4148323 G allele acted synergistically to increase the risk of laryngeal cancer. In the present study, higher MAF of rs4148323 in our cohort suggest that the alleles could be associated with the pathogenesis of laryngeal squamous cell carcinoma.

Genetic variants and environmental factors are two important factors associated with carcinogenesis. Several studies have shown that DNA polymorphisms and smoking or alcohol consumption play an important role in the occurrence of laryngeal cancer [23,24,25]. Cigarette smoke carcinogens and alcohol consumption produce large amounts of reactive oxygen molecules, causing double-stranded DNA breaks that can lead to tumor formation and development [26,27]. UGT1A1 has been reported to be important in the detoxification of tobacco smoke carcinogens [5]. UGT1A1 is also the only enzyme which catalyzes the glucuronidation of the potent antioxidant bilirubin, which may play a protective role against cancer [10,11,12]. rs4148323 A allele is independently associated with increased total bilirubin levels [7,18]. Since serum bilirubin concentrations are inversely correlated with UGT1A1 activity and the rs4148323 A allele is associated with decreased UGT1A1 activity, it can hypothesized that rs4148323 A allele might decrease individual risk of developing laryngeal cancer. In this study, we have found that rs4148323 G allele was associated with laryngeal cancer. Laryngeal cancer is the second most common type of head and neck tumor, and the most common histological type is squamous cell carcinoma. Our results showed that smoking and alcohol consumption were risk factors of laryngeal carcinomas, which is consistent with previous studies [28,29]. Over one third of laryngeal cancer patients present with advanced stage III and IV disease at the time of diagnosis [30]. This might be one of the important reasons affected the prognosis of the disease. It is important to quit smoking, control alcohol consumption for prevention the occurrence of laryngeal cancer. In addition, it is necessary to identifying biomarkers for screening laryngeal cancer risk. Thus, an improved understanding of the underlying molecular mechanisms is required to facilitate the development of more effective diagnostic and therapeutic strategies. Recently, a number of new molecular markers have been found successively, ERCC1 rs11615 and ERCC5 rs17655 polymorphisms [31], genetic variation of MT2A [32], CYP1B1*2 355T and CYP2E1*5-1293C [33] and XPG Asp1104His [34] were reported to be associated with increased risk of laryngeal cancer, while ERp57-STAT3 regulation functions may regulation functions in radioresistance of laryngeal cancer, and targeting the ERp57-STAT3 pathway might be important for enhancing the efficacy of radiotherapy in human laryngeal cancer [35].

Our study focused on the relation between the rs4148323 A/G polymorphism and the risk of laryngeal cancer and the polymorphisms were detected in all subjects at the time of diagnosis. Next we will expand sample size to further confirmation of the relation between polymorphism and the occurrence of laryngeal cancer. In addition, follow-ups will be given to the cohort to observe the prognosis of different genotypes after treatment. Confirmation of such a correlation might provide a new biomarker to predict the risk of laryngeal cancer.

## 5. Conclusions

In conclusion, the rs4148323 G allele is associated with the high UGT1A1 enzyme activity, and may increase the risk of laryngeal cancer. Besides, smoking or alcohol consumption and the rs4148323 G allele act synergistically to increase the risk of laryngeal cancer. The impact of UGT1A1*6 polymorphisms in relation to laryngeal cancer risk requires further validation in studies including larger samples. Follow-ups to the cohort are also important to analyze the prognosis of different genotypes after treatment.
